# Novel Methodology to Assess Salt Movement Between Mortar and Stones from Heritage in Spain

**DOI:** 10.3390/ma18143340

**Published:** 2025-07-16

**Authors:** Linde Pollet, Andrea Antolín-Rodríguez, Josep Gisbert-Aguilar, Gabriel Búrdalo-Salcedo, Andrés Juan-Valdés, César García-Álvarez, Angel Raga-Martín, Wouter Schroeyers, Víctor Calvo, María Fernández-Raga

**Affiliations:** 1 European Commission, Joint Research Centre (JRC), 2400 Geel, Belgium; 2 Research Group NuTeC, Centre for Environmental Sciences (CMK), Hasselt University, 3590 Diepenbeek, Belgium; wouter.schroeyers@uhasselt.be; 3 Department Engineering and Agricultural Sciences, Universidad de León, 24004 León, Spain; aantr@unileon.es (A.A.-R.); ajuav@unileon.es (A.J.-V.); 4 Department of Geology, Universidad de Zaragoza, 50009 Zaragoza, Spain; gisbert@unizar.es; 5 Department of Mechanical Engineering, Computer Science, and Aerospace, Universidad de León, 24004 León, Spain; gabriel.burdalo@unileon.es; 6 Department of Artistic and Documentary Heritage, Universidad de León, 24004 Léon, Spain; cgara@unileon.es; 7 Department of Applied Physics and Chemistry, Universidad de León, 24004 León, Spain; aragam00@estudiantes.unileon.es; 8 Instituto de Carboquímica (ICB-CSIC), 50018 Zaragoza, Spain; vcalvo@icb.csic.es

**Keywords:** alkali-activated materials, salt movement, conductivity, construction, sustainability

## Abstract

The development of sustainable cementitious materials is crucial to reduce the environmental footprint of the construction industry. Alkali-activated materials (AAMs) have emerged as promising environmentally friendly alternatives; however, their compatibility with natural stone in heritage structures remains poorly understood, especially regarding salt migration and related damage to stones. This study presents a novel methodology for assessing salt movement in solid materials between two types of stones—Boñar and Silos—and two types of binders: blended Portland cement (BPC) and an AAM. The samples underwent capillarity and immersion tests to evaluate water absorption, salt transport, and efflorescence behavior. The capillarity of the Silos stone was 0.148 kg·m^−2^·t^−0.5^, whereas this was 0.0166 kg·m^−2^·t^−0.5^ for the Boñar stone, a ninefold difference. Conductivity mapping and XRD analysis revealed that AAM-based mortars exhibit a significantly higher release of salts, primarily sodium sulfate, which may pose a risk to adjacent porous stones. In contrast, BPC showed lower salt mobility and different salt compositions. These findings highlight the importance of evaluating the compatibility between alternative binders and heritage stones. The use of AAMs may pose significant risks due to their tendency to release soluble salts. Although, in the current experiments, no pore damage or mechanical degradation was observed, additional studies are required to confirm this. A thorough understanding of salt transport mechanisms is therefore essential to ensure that sustainable restoration materials do not inadvertently accelerate the deterioration of structures, a process more problematic when the deterioration affects heritage monuments.

## 1. Introduction

Cement production accounts for up to 8% of global CO_2_ emissions, highlighting the urgent need for more sustainable construction practices. A circular economy requires designing new, clean, and sustainable products and materials with reduced environmental impact [1]. In addition, it is essential to preserve for the future the usefulness, economic maintenance, properties, and protection of currently available resources [2].

The development, use, and application of alternative construction materials, such as alkali-activated materials (AAM), is widely researched [3,4]. They are a class of inorganic binders produced by reacting aluminosilicate materials with an alkaline activator. Industrial residues are often used as aluminosilicate source, which lowers their environmental impact. The compressive strength of these materials varies between 15 and 80 MPa, depending on the materials used [5,6], and in some cases goes up to 122 MPa [7]. Since AAMs are made with an alkaline solution, they present new challenges, including the risk of salinization, which is currently one of the most significant causes of structural damage in infrastructure [8,9]. A notable example of this phenomenon is the “mal de la piedra” (an approximate translation would be ‘stone disease’), a form of deterioration which affects various types of limestone commonly used in monuments and sculptures [10,11,12]. The condition arises from a combination of physical and chemical processes. Moisture, often in the form of rainwater or ambient humidity, infiltrates the porous surface of the stones. This causes salts to dissolve that are either naturally present or introduced through environmental pollution [13]. Acid rain contains SO_4_^2−^, an ion that can cause severe damage in the form of salt weathering [14]. As the moisture evaporates, these salts crystallize within the pores, creating internal pressure that gradually damages the mineral structure [15,16]. As a result of climate change conditions, damage by wetting in infrastructure is increasing, exacerbated by salt movement [17,18,19]. Salts are transported with the water into the pores of the stone. During dehydration, these salt crystals cause stress and changes in the micropores, increasing stone deterioration [20,21]. Additionally, temperature fluctuations increase internal pressure, accelerating stone deterioration. This translates into severe consequences for the stonework of historic monuments and structures in cities with high humidity, high water table, and sudden temperature changes, as in cities located in the north of Spain such as León [17]. The salt deposited in the pores is hydrophilic and thus attracts environmental change which causes the surface of the stone to expand over time, leading to aesthetic damage [18,22]. The pores gradually increase in size and depth, joining together to form a pore gallery and therefore lose intermediate material. This ultimately causes sudden wear and loss of material progressing from the surface to the interior and eventually affecting the internal structure of the stone [9]. As a consequence, inscriptions in monuments gradually fade, deterioration expands deeper into the stone, and the loss of cohesion can ultimately lead to partial or complete collapse. Understanding and mitigating these processes is, therefore, essential for both the development of sustainable materials and the preservation of cultural and architectural heritage.

Since no definitive solution to this issue is currently available, the impact of using mortars with reduced salt production must be assessed to avoid the enrichment of stones with salt and the exacerbation of the salinization problem. However, restoration work on stone monuments has not always been performed by companies aware of this issue. Often, due to a lack of knowledge, resources, or interest, these companies use mortars that are familiar to them (such as Portland cement) or easier to use, despite the fact that different types of mortars have been identified as possible causes of salt mobilization from the mortars to the monument’s stones [19]. Nowadays, when selecting mortar for restoration works, factors such as future performance, evolution within the monument, or potential environmental impact should be considered [23]. The main objective is to find a suitable, functional mortar that will not damage the stone monument and is both inexpensive and easy to apply. An added bonus would be to use mortar recycled from residue materials from other activities, as this would be more sustainable and affordable. Since Portland cement is the most widely used mortar type in the world, we need to be able to evaluate its impact on different stone substrates, as well as to compare its effectiveness regarding salinization after continuous wetting–drying cycles with that of mortar produced from industrial residues, such as AAMs. The overarching objective is to develop environmentally sustainable mortar formulations which are not only functional and cost-effective but also compatible with historic stone. Ensuring that these binders do not contribute to salt migration or accelerate material degradation is fundamental to their long-term viability in heritage conservation.

The objective of this study is to develop and apply a novel methodology to qualify salt movement through conductivity measurements between cement and stone on solid surfaces. This study investigates whether it is possible to modify a conductivity meter to qualitatively assess changes in the surface conductivity of solid surfaces. We analyzed two different stones (Boñar stone and Silos stone) in combination with blended Portland cement (BPC) and an AAM from industrial residues. To do so, capillarity and immersion tests were performed on the samples to evaluate the ability of a liquid to penetrate the materials through their pores and to assess salt mitigation between Boñar/Silos stones and the different cementitious materials used.

## 2. Materials and Methods

### 2.1. Experimental Materials

#### 2.1.1. Mortars

Four different mortar mixtures were prepared for the work: BPC with sand, AAM with sand, BPC without sand, and AAM without sand.

The BPC used here is a CEM II/B-M type with fly ash and limestone (V-L) additives, exhibiting a 28-day compressive strength of 32.5 MPa. Its chemical composition complies with the requirements of EN 197-1 [24]. AAM was synthesized from industrial by-products. The precursor mix consisted of 30 wt% phosphogypsum (PG) and 70 wt% ground granulated blast furnace slag (GGBFS). The GGBFS was derived from a Dutch steel industry and conforms to EN 15167-1 [25]. The PG was dried at 40 °C and milled to a particle size smaller than 250 µm. Their main composition is cited in Table 1.

Tap water was used in the preparation of BPC mortars, while AAM ones were activated using a 4 M NaOH solution prepared with distilled water 24 h in advance and added gradually during mixing.

The sand used in the mixtures is commercially available standardized sand that conforms to the specifications established in EN 196-1 [26]. It was obtained by screening natural quartz-rich sands. This sand is characterized primarily by its particle size distribution, with grain sizes ranging from 0.08 to 2.00 mm. Mortar formulations follow the guidelines of EN 196-1. Sand mortars were prepared using a binder-to-sand ratio of 1:3 and a liquid-to-binder ratio (water or NaOH solution) of 1:2. Binder-only mortars were formulated with a liquid-to-binder ratio of 1:2.

#### 2.1.2. Stones

Two types of stone were used for the present work: Boñar stone and Silos stone. Their primary difference is mineralogical composition. Boñar stone consists of 87 wt% dolomite, 12 wt% calcite, and 2 wt% silica, whereas Silos stone is composed of 90 wt% dolomite, 9 wt% calcite, and 1 wt% silica.

### 2.2. Sample Preparation

The mixing procedure for sand mortars was as follows: the liquid component (either tap water or NaOH solution) and the corresponding binder were first introduced into the mixing bowl. The mixer was operated at low speed for 30 s while gradually adding the sand over the next 30 s. Mixing continued for an additional 30 s at high speed. The mixer was then stopped for 90 s, followed by another 60 s of mixing at high speed. The missing procedure for binder-only mortars was the same but omitting the addition of sand and its corresponding 30 s mixing time.

**Figure 1:**
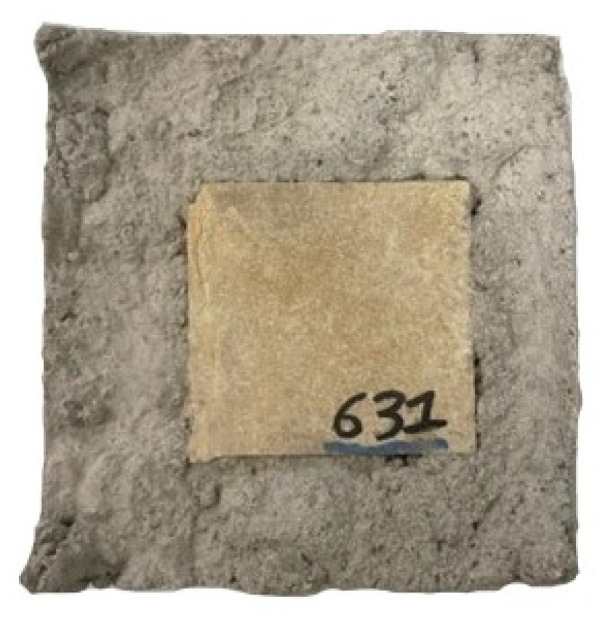
Spatial configuration of samples.

After mixing, the mortar was transferred to a vibration table to eliminate entrapped air and then cast into cubic molds (100 mm × 100 mm) containing the stone samples centrally embedded. The spatial configuration of the samples for testing is illustrated in Figure 1. This sample setup was used for both capillary absorption and immersion tests.

A summary of the final configuration of the specimens used in the various experimental procedures is presented in Table 2, considering both the type of stone and the surrounding matrix material.

### 2.3. Methods

#### 2.3.1. Capillarity Tests

Capillarity plays a crucial role in transport dynamics within porous materials; we assess it by measuring water absorption through one face of each sample, partially submerging it (2 mm) in distilled water using a saturated cloth placed in a tray. All lateral surfaces were sealed with plastic film to prevent lateral evaporation. Mass measurements were taken at regular intervals (0.5, 1, 2, 3, 5, 7, 10, 15, 20, 30, 40, 45, 60, 90, and 120 min), followed by measurements at 8 and 24 h, though no significant changes were observed beyond 120 min.

This test was conducted on both bare stone samples and composite specimens containing embedded stone and mortar. Each composite sample was dried and reused for replication in two additional cycles.

The absorbed mass per surface area unit was plotted against the square root of time to calculate the capillarity coefficient (*C*) from the slope of the fitted curve. Of note, AAM-based specimens displayed salt efflorescence upon drying, which was subsequently analyzed using X-ray diffraction (XRD). In addition, XRD measurements were performed on each type of stone and mortar, as well as on the salts detected after the experiment, using a Bruker D-8 Advance diffractometer from Karlsruhe, Germany with Bragg–Brentano geometry in the 2θ range of 15° to 80°, with a step size of 0.03°. Prior to analysis, the samples were manually ground in an agate mortar to reduce particle size and improve homogeneity, thereby minimizing absorption effects related to differences in the mass absorption coefficients of the present phases. Phase identification was performed by comparing the experimental patterns with reference data from the ICDD-PDF2 database using BRUKER Diffrac.Eva V5.2. Quantitative phase analysis was carried out through Rietveld refinement using BRUKER Diffrac.Topas V6.0, employing crystal structure data from the ICSD database.

#### 2.3.2. Conductivity Meter Modification and Calibration, and Conductivity Measurement on Solid Surfaces

**Figure 2:**
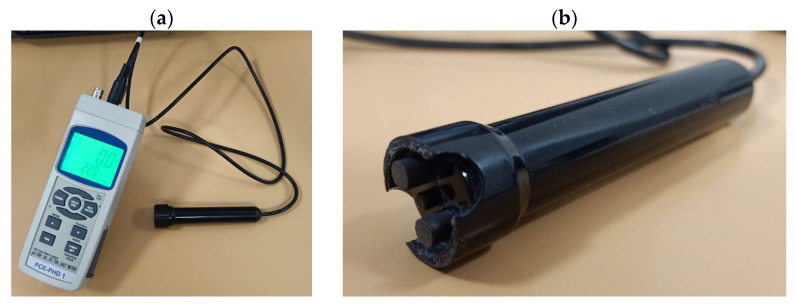
(**a**) Conductivity meter PCE-PHD 1 from PCE Instruments with (**b**) modified conductivity sensor.

Surface conductivity is used to assess salt content and movement in solid samples. The conductivity meter used in this work is a PCE-PHD 1 model from PCE Instruments (Figure 2a), equipped with an external measuring sensor (Figure 2b). Since a conductivity meter is designed to measure liquid, the sensor was modified to enable measurements on solid surfaces. This modification consisted in removing two electrodes from the sensor head and placing them in a completely horizontal position, so that the temperature sensor, the current transmitter, and the receiver can come into contact with a flat surface, with gold as the conductive metal. By doing so, measurements can be taken directly on a solid surface using simple drops of water and can still be taken on liquids as previously achieved.

**Figure 3:**
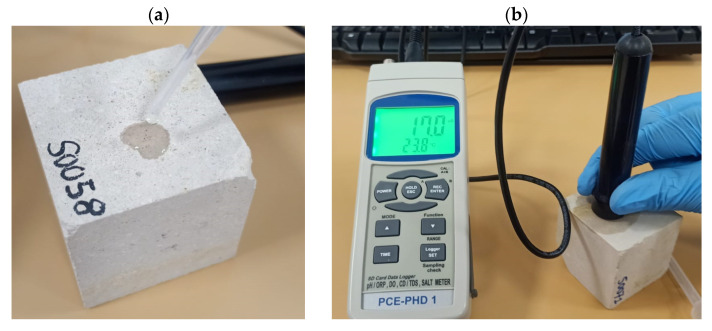
Conductivity measurement on Silos stone. (**a**) Additions of a drop distilled water; (**b**) measurement.

We obtained measurements on solid surfaces by placing two drops of distilled water on the sample using a Pasteur pipette and placing the sensor above them perpendicularly to the surface (Figure 3), ensuring both electrodes were in contact with the wet surface. Each wet surface area was isolated from the previously measured areas by ensuring the drops not touching. This allows for the comparison of measurements between samples. Since distilled water is extremely poorly conductive (2 ± 0.5 µS), changes in surface conductivity must result from variations in salt concentration on the solid surface.

#### 2.3.3. Modified Conductivity Sensor Calibration

The results obtained using the novel method explained above slightly differ from those obtained in submerged solutions. Therefore, our conductivity measurements can be used to compare samples as long as the same method is employed, but these measurements cannot be used directly to compare samples with those whose conductivity was measured in solution. The conductivity measurements taken will allow us to assess, in a more qualitative way, changes in the surface conductivity. To reach this conclusion, we conducted a preliminary calibration phase in which measurements taken on a stone immersed in water were compared with those taken on a stone with a few drops of water on its surface.

The aims of this calibration phase are to verify that our modification did not alter the meter’s capabilities and that the measurements obtained following our novel method are similar to those obtained following the traditional method, for which a HI98127 liquid conductivity meter (HANNA Instruments, from Nusfalau, Salaj county in Romania) was used. Tests were performed following the traditional method on different solutions: a mixture of distilled water with AAM powder, a mixture of distilled water with BPC powder, and distilled water alone. We expected similar results from both conductivity meters.

**Figure 4:**
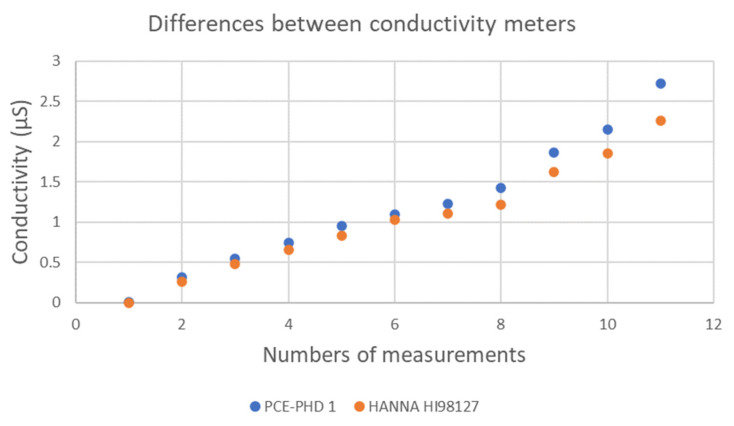
Comparison between the not-modified conductivity meter (HANNA HI98127) and the modified one (PCE-PHD 1).

The measurements obtained using the modified conductivity meter and those obtained using the unmodified HANNA conductivity meter yielded very similar results (Figure 4), with a maximum variability of just 2% for pH values below 8 and a maximum difference of 18% for pH values above 8. These surface conductivity measurements can only provide a qualitative indication of the salt content on the surface of the samples.

#### 2.3.4. Natural Stone Surface Conductivity

To assess salt mitigation, we measured the surface conductivity of the natural Boñar and Silos stones and mortars was measured individually so that changes in conductivity could later be determined. Each surface was measured using the modified conductivity meter on nine (3 × 3) measurement points separated by 2 cm. To correct for the variation between stones of the same type, this process was repeated on three Boñar and three Silos stones, for which surface conductivity maps were later created (Appendix A). The average surface conductivity was calculated and integrated into one surface conductivity map for each type of stone. In addition, surface conductivity was also measured in AAM and BPC samples, and these results were also used to create surface conductivity maps.

#### 2.3.5. Immersion Tests

To assess salt transfer between the cementitious materials and the stones, the samples were immersed in a basin of distilled water, allowing the movement of salts to occur. Only samples containing the same type of mortar (BPC or AAM) were placed together. The immersion water was replaced between batches. However, in some cases, mortar did not stick sufficiently to the samples when removing these from the molds. Therefore, not all samples could be used in this test. The effect of water on the stones can also be illustrated. After the samples were submerged for 2 days, they were turned over and kept submerged for another 2 days. After 4 days of submersion, samples were dehumidified in a vacuum-drying oven until their original mass was reached (assuming no significant mass loss). Thorough monitoring was conducted during the drying phase for stone samples without mortar to model the time required to dry completely. When drying the stones with mortar in the vacuum-drying oven, these were turned over halfway through the drying process to neutralize the effect that gravity might have had on the salt movement, so that salt migration could be uniform across the entire surface and in all directions. The conductivity of the mortar/stone samples was measured with the modified conductivity meter after they were submerged and dried. Surface conductivity maps were created with these results; stone surface is marked with a red rectangle in these maps.

## 3. Results and Discussion

### 3.1. Capillarity

The water absorption capacity of the stones was assessed prior to the capillarity test. Silos stone presents a higher water absorption capacity than Boñar stone. In same size samples (5 × 5 × 5 cm), Boñar stone absorbs approximately 10 g of distilled water, while Silos stone absorbs around 33 g. This capacity was later modified by repeated tests with the mortar applied.

**Figure 5:**
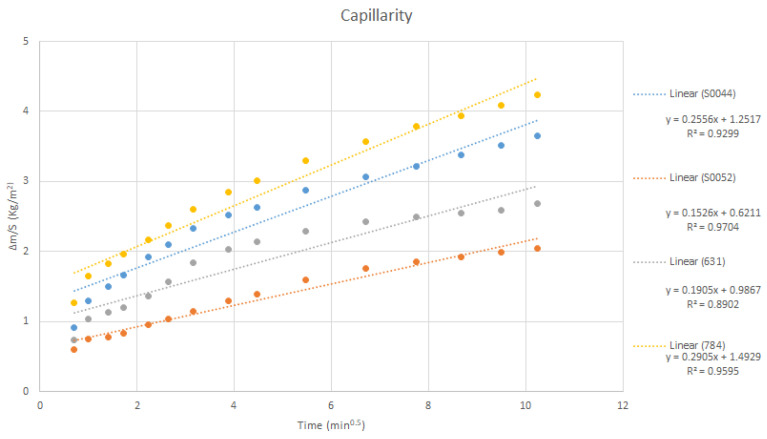
Fit of capillarity results for Boñar and Silos stones.

The change in weight per surface area was plotted throughout the experiment (square root of the time); the slope represents the capillarity coefficient (Figure 5). Capillarity tests of the stones showed that Silos stones present higher capillarity compared with Boñar stones. Silos stone capillarity was, on average, 0.148 ± 0.063 kg·m^−2^·t^−0.5^; Boñar stone capillarity was, on average, 0.0166 ± 0.002 kg·m^−2^·t^−0.5^. The difference is likely due to a higher porosity and lower density of the former [27,28,29]. When conducting this test on the stones with mortar (Table 3), it is noticeable that the samples performed similarly during the different repetitions, except for sample S0052 (made with BPC and without sand).

Results suggest a moderate increase in capillarity when either binder is combined with Silos stone compared with Boñar stone, likely due to the former’s higher porosity. Notably, BPC mortars exhibited initially lower capillarity than AAM mortars, but this value increased upon repeated testing, indicating a potential structural evolution or enhanced moisture uptake over time. In contrast, AAM samples showed consistent capillarity across repeated cycles, suggesting greater structural stability under capillary stress.

The XRD analysis of the formed salts during the experiment revealed that the salts collected from the samples prepared with AAM consisted primarily in Na_2_SO_4_, with a concentration of up to 85%. No significant difference was appreciated in the composition of salts, regardless of whether these were collected from the stone or the AAM. Conversely, salts collected from the samples with BPC consisted in Al_2_O_3_. Additionally, calcite and dolomite phases were also present in the salt samples from the cementitious materials, originating directly from the stones.

**Figure 6:**
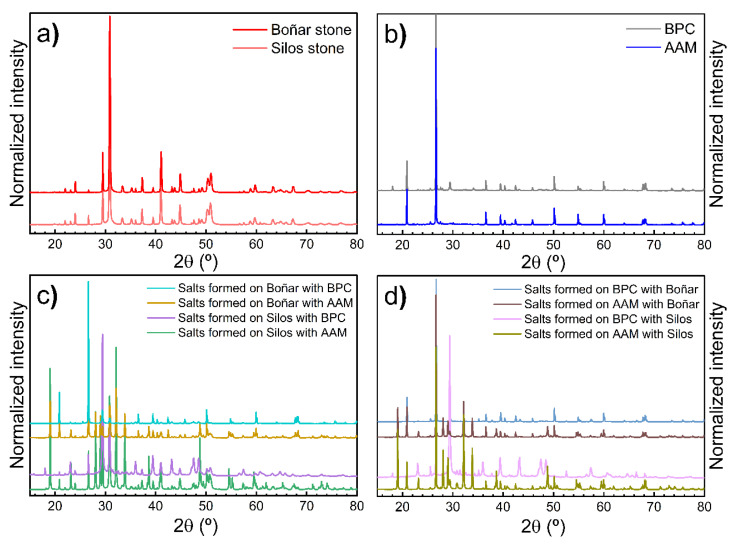
Representative XRD patterns of (**a**) the stones, (**b**) the mortars, (**c**) the salts formed on the stones, and (**d**) the salts formed on the mortars.

The crystalline phases present in the stones, mortars, and emerged salts were analyzed by XRD (Figure 6). Their corresponding quantified percentages of each phase are detailed in Table 4. Both Silos and Boñar stones show similar diffractograms (Figure 6a), with a composition dominated by dolomite and calcite, confirming their classification as dolomitic limestones. The mortars (Figure 6b) are rich in quartz, with clear compositional differences between BPC and AAM. BPC contains alite, larnite, and lime, whereas AAM presents additional phases such as orthoclase, oligoclase, muscovite, and ettringite, indicating a more heterogeneous nature.

Carbonate phases (dolomite and calcite) and quartz are present in all salt samples (Figure 6c,d), likely due to a partial dissolution and reprecipitation of the stone material during the salt crystallization processes. Thenardite (Na_2_SO_4_) is highly present in salt samples in contact with AAM, with contents reaching up to 60%, regardless of whether they formed on the stone or the mortar. In contrast, salts associated with BPC showed a lower sulfate content and the presence of larnite in some of them. Notably, corundum (Al_2_O_3_) appears in salts from BPC systems in contact with both stones. Overall, the combination of the XRD patterns (Figure 6) and the quantitative data (Table 4) provides a comprehensive understanding of the crystallization behavior of the salts, strongly influenced by both the type of mortar and the nature of the substrate.

### 3.2. Surface Conductivity Before and After Immersion

**Figure 7:**
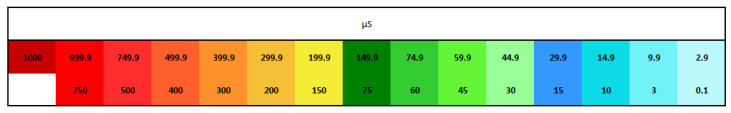
Scale used in surface conductivity maps with the mean value of each category.

Surface conductivity measurements enabled the drawing of maps to visualize changes in surface conductivity and, thus, in the amount of salt present on the surface of the different samples. Figure 7 shows the color code scale applied in the surface conductivity maps.

#### 3.2.1. Boñar Stone Samples

**Figure 8:**
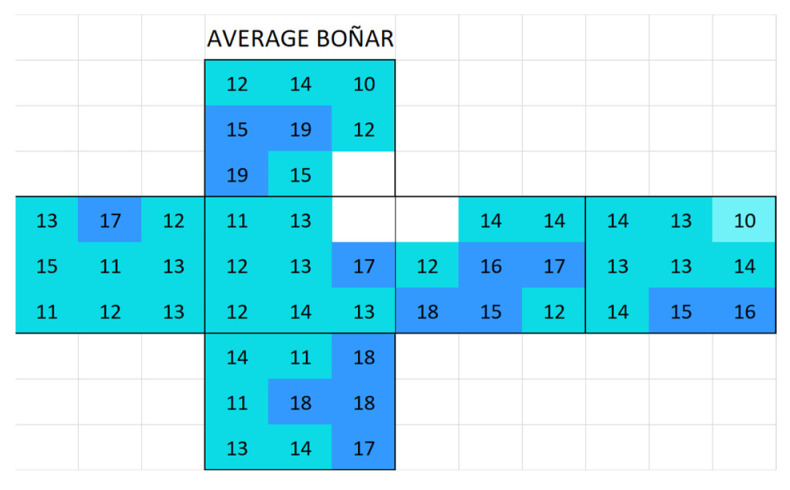
Map of the average surface conductivity of natural Boñar cubic stones without any contact with mortar in any of the six faces.

**Figure 9:**
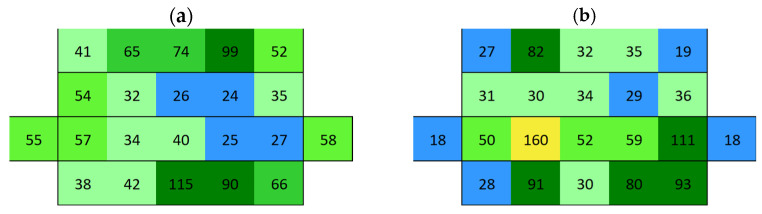
Map of average surface conductivity on a prismatic mortar made of (**a**) BPC without sand and (**b**) BPC with sand.

**Figure 10:**
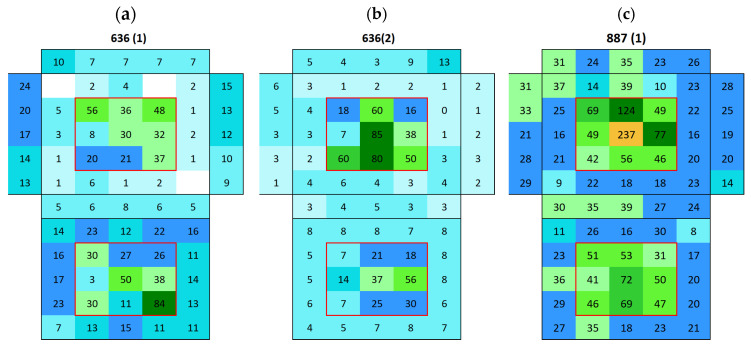
Surface conductivity maps of Boñar stone with BPC sample (cubic stone surrounded by mortar): (**a**) 636 after first immersion, (**b**) 636 after second immersion, and (**c**) 887 after first immersion.

Boñar stone showed an average surface conductivity of 13.9 ± 2.4 µS (Figure 8). The average distribution of surface conductivity in BPC with and without sand is shown in Figure 9. An increasing movement of salts from mortar to stone was observed in sample 636 (Boñar with BPC, Figure 10a) after immersion; over the two immersion cycles, the surface conductivity of the Boñar stone increased (within the red rectangle), while that of the BPC decreased (outside the rectangle), compared with the value of the natural materials (Figure 10b). After measuring the surface conductivity of sample 887 (Figure 10c) following the first immersion test, for which the only difference was the absence of sand in the mortar, the overall surface conductivity was higher, both for the stones and mortar, with the highest conductivity measured (237 μS) at the center of the stone at the front. However, an increase in conductivity in this range is less expected in samples with BPC, since the average surface conductivity of BPC before immersion is 52 ± 36 µS with sand and 52 ± 24 µS without sand.

**Figure 11:**
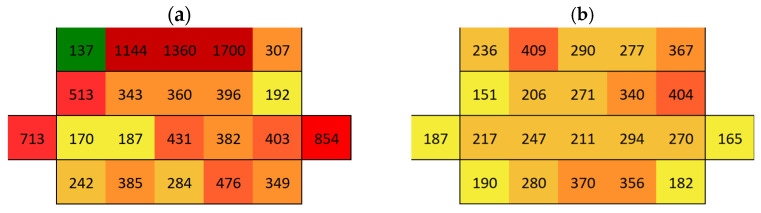
Map of average surface conductivity on a prismatic mortar: (**a**) AAM without sand and (**b**) AAM with sand.

**Figure 12:**
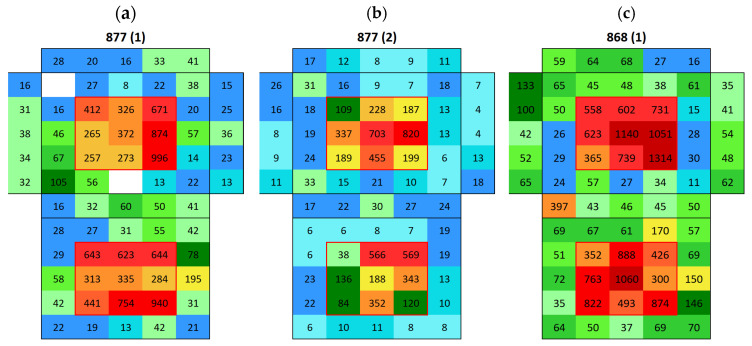
Surface conductivity maps of the AAM with Boñar stone sample (cubic stone surrounded by mortar): (**a**) 877 after first immersion, (**b**) 877 after second immersion, and (**c**) 868 after first immersion.

The surface conductivity and, thus, the salt content on AAM after the immersion of samples 877 and 868 was of the same order of magnitude, regardless of whether AAM was made with or without sand. However, salt content on the surface of Boñar stone was 21% higher when sand was mixed within the AAM compared with when it was not. When analyzing the materials separately, the AAM without sand exhibited higher surface conductivity (on average 515 µS, Figure 11a) than AAM with sand (on average 269 µS, Figure 11b). The presence of sand affects surface conductivity in AAM, as adding sand to the mortar results in a mixture containing less NaOH for the same volume, influencing the formation of Na_2_SO_4_, which is the most commonly formed salt in this type of mortar. During the hardening process of the AAM around the Boñar stone, it appears that the majority of salts from the AAM mixture seem to have moved to the Boñar stone instead of curing in the AAM matrix. After the first immersion of sample 877 (Boñar stone with AAM without sand, Figure 12a), a higher conductivity was obtained on the surface of the Boñar stone (in the red rectangle, on average 523 μS) compared with that of the AAM (outside the rectangle, on average 37 μS). The second immersion caused both conductivity values to decrease on average to 312 μS for the Boñar stone and 14 μS for the AAM (Figure 12b). Following the first immersion (Figure 12c), sample 868 exhibited a surface conductivity of 634 μS on the Boñar stone (central area inside the red rectangle), significantly higher than the 39 μS measured on the surrounding AAM (outside red rectangle). This disparity strongly indicates substantial salt migration from the AAM to the stone substrate during wetting–drying cycles.

#### 3.2.2. Silos Stone Samples

**Figure 13:**
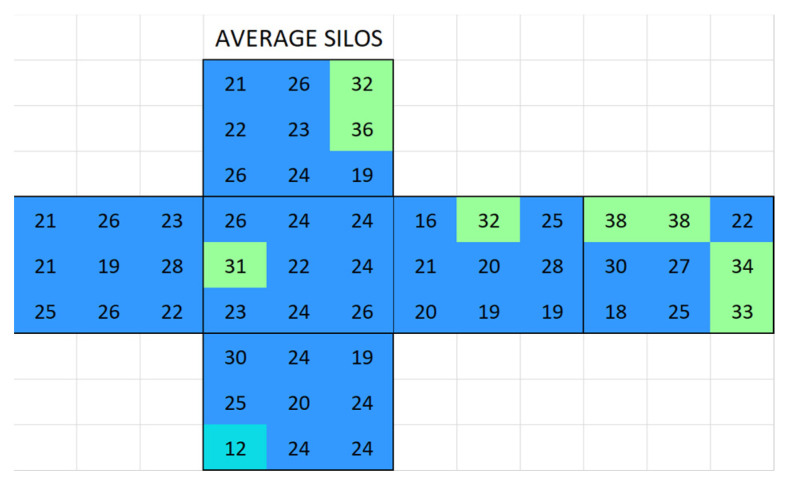
Map of the average surface conductivity of natural Silos cubic stone without any contact with mortar on any of the six faces.

**Figure 14:**
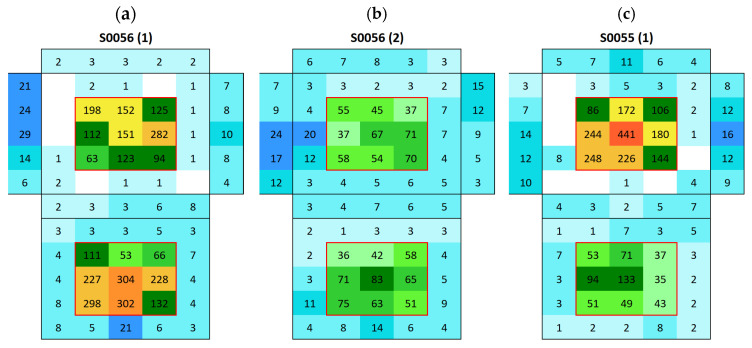
Surface conductivity maps of BPC with Silos stone samples (cubic stone surrounded by mortar): (**a**) S0056 after first immersion, (**b**) S0056 after second immersion, and (**c**) S0055 after first immersion.

**Figure 15:**
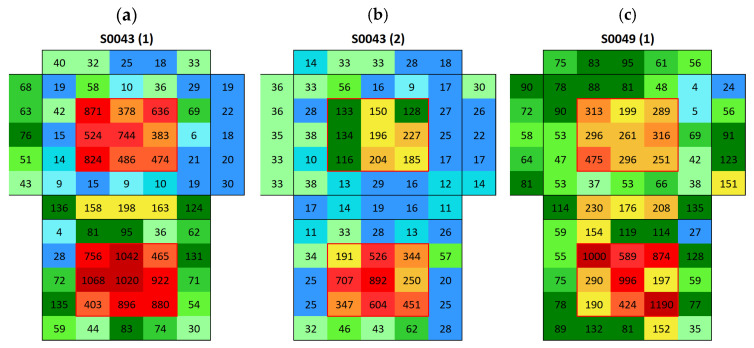
Surface conductivity maps of AAM with Silos stone samples (cubic stone surrounded by mortar); (**a**) S0043 after first immersion, (**b**) S0043 after second immersion, and (**c**) S0049 after first immersion.

Silos stone showed an average surface conductivity of 24.6 ± 5.2 µS, which is significantly higher than that of the Boñar stone. The average distribution is shown in Figure 13. After immersing the samples made with Silos stone and BPC (Figure 14a,c), a higher surface conductivity was obtained on the surface of both Silos stones, with an average of 168 μS (sample S0056) and 133 μS (sample S0055), compared to the low conductivity of the individual materials. A lower salt content was observed in the stone in samples with BPC with sand than in those with BPC without sand. Surface conductivity measured on the surface of the mortar was lower after immersion, indicating salt movement from BPC to the Silos stones. Still, the difference between values corresponding to BPC with sand and BPC without sand was almost negligible, with an average of 5 μS and 6 μS, respectively. A second immersion of the sample (Figure 14b) resulted in a 69% decrease in the surface conductivity of the Silos stone with no increase in the conductivity of the surrounding material. This suggests that the salt on the surface was washed out, potentially due to the dissolution of the salts in the water during the immersion. Further analysis is needed to monitor the conductivity of water during immersion tests and to assess whether or not the formed salts dissolve in water. After the second immersion, fewer salts were formed compared with those after the first one. The same occurred when AAM was combined with a Silos stone (Figure 15). Both samples, S0043 and S0049, were prepared identically; however, surface conductivity after the first immersion significantly differed between them; surface conductivity was lower in both samples after the second immersion.

In general, the surface conductivity of samples made with Silos stones was higher after the first immersion than that of samples made with Boñar stones. This correlates with the significantly higher capillarity of Silos stones compared with that of Boñar ones and thus also with a higher affinity for the intrusion of humidity potentially with the presence of dissolved salts. The repetition of the immersion test allows the initially deposited salts to wash off from the surface. Repetition is needed to assess further washout.

## 4. Conclusions

Here we present a novel methodology using a modified conductivity meter, which qualitatively assesses and maps salt migration between mortar and stone surfaces, offering a significant advancement for heritage conservation monitoring. Two types of cementitious materials were tested in combination with two types of stones commonly used in Spanish heritage structures. Our findings critically reveal that alkali-activated materials (AAMs) exhibit a higher propensity for sodium sulfate salt release, posing a considerable risk for the accelerated deterioration of adjacent porous stones, particularly compared to modified Portland cement (BPC). Capillarity and immersion tests were performed on the different samples. The capillarity of the Silos stone was, on average, 0.148 kg·m^−2^·t^−0.5^, whereas this was 0.0166 kg·m^−2^·t^−0.5^ for the Boñar stone, which is a 9-fold difference. Immersion tests revealed an initial movement of salts from cementitious materials to stones, which was partially washed away when samples were re-immersed. XRD analysis of the salts revealed the formation of Na_2_SO_4_ on surfaces of samples made with AAM, whereas, on samples made with BPC, this was Al_2_O_3_. Calcite and dolomite phases were found in the collected salts on the surface of all the samples, originating directly from the stones.

We tested our novel approach to surface conductivity measurement on solid samples to indicate the presence and movement of salts, the most significant issue detected in carbonated stones. Surface conductivity measurements revealed AAM-based mortars to be associated with significantly higher salt release compared with BPC formulations. This elevated salt migration, predominantly in the form of sodium sulfate, raises concerns regarding the compatibility of AAM with porous carbonate stones in heritage applications. These results emphasize the necessity of pre-evaluating the interactions between new binders and historic substrates to ensure both environmental sustainability and material preservation.

The immersion tests revealed an initial movement of the salts from the cementitious materials to the stones, which was partially washed away when the samples were re-immersed. XRD analysis of the salts revealed the formation of Na_2_SO_4_ on the surface of the samples made with AAM, whereas, on the samples made with BPC, this was Al_2_O_3_. Calcite and dolomite phases were found in the collected salts on the surface of all samples, originating directly from the stones.

By synthesizing data on capillarity and conductivity, this work provides a deeper understanding of the complex interactions between different mortar types and stone substrates, underscoring the critical need to pre-evaluate material compatibility to ensure long-term preservation. Consequently, this research emphasizes the imperative for developing restoration mortars that are not only sustainable but also fully compatible with historical materials, thereby mitigating salt-induced degradation and guiding future efforts in heritage conservation. Further optimizing conductivity measurements is necessary to enable the quantitative assessment of salt movement between mortars and stones before these can be applied in real-world applications.

## Figures and Tables

**Table 1:** Main elemental composition (in wt%) of PG and GGBFS.

	Na	Mg	Al	Si	P	S	Ca	Sr
PG	4.6	-	0.8	-	0.21	18	21	1.6
GGBFS	1.2	7.8	9	25	-	1.3	38	0.1

**Table 2:** Overview of used samples for the different experiments, with the stone numbers, and in brackets, with or without sand.

	Boñar Stone		Silos Stone	
	BPC	AAM	BPC	AAM
Capillarity	631 (with)	784 (with)	S0052 (without)	S0044 (with)
Immersion	636 (with)	868 (with)	S0055 (with)	S0043 (without)
	887 (without)	877 (without)	S0056 (without)	S0049 (without)

**Table 3:** Capillarity coefficient experimental results for the stone cementitious material samples.

	Boñar Stone		Silos Stone	
	BPC	AAM	BPC	AAM
	631 (with sand)	784 (with sand)	S0052 (without sand)	S0044 (with sand)
1st experiment	0.176	0.232	0.459	0.369
2nd experiment	0.181	0.291	0.153	0.256
3rd experiment	0.275	0.281	0.790	0.346
Mean	0.211	0.268	0.467	0.324
StDev	0.056	0.032	0.319	0.060

**Table 4:** Quantitative XRD results showing the percentage of crystalline phases identified in the different samples analyzed: stones, mortars, and salts formed.

	Dolomite MgCa(CO_3_)_2_	Calcite CaCO_3_	Quartz SiO_2_					
Boñar stone	93.122	6.471	0.408					
Silos stone	87.899	11.668	0.433					
	Dolomite MgCa(CO_3_)_2_	Quartz SiO_2_	Orthoclase KAlSi_3_O_8_	Oligoclase (Ca, Na) (Al, Si)_4_O_8_	Alite Ca_3_SiO_5_	Muscovite KAl_2_(AlSi_3_O_10_) (F,OH)_2_	Ettringite Ca_6_Al_2_(SO_4_)_3_ (OH)_12_·26H_2_O	Lime Ca(OH)_2_
BPC	16.744	65.535	5.866	1.101	5.449			5.303
AAM		68.863	11.206	1.865	4.098	2.393	11.574	
	Dolomite MgCa(CO_3_)_2_	Calcite CaCO_3_	Quartz SiO_2_	Larnite Ca_2_SiO_4_	Corundum Al_2_O_3_	Thenardite Na_2_SO_4_	Anhydrite CaSO_4_	Lime Ca(OH)_2_
Salts formed on Boñar with BPC	16.542	28.034	52.806	2.347			0.271	
Salts formed on Boñar with AAM	26.35	7.598	21.328			44.723		
Salts formed on Silos with BPC	32.761	59.028	1.57	4.861				1.78
Salts formed on Silos with AAM	35.824	6.682	3.193			54.301		
Salts formed on BPC with Boñar		23.646	61.394		14.96			
Salts formed on AAM with Boñar		9.64	25.77			60.125	4.465	
Salts formed on BPC with Silos	2.287	81.389	1.358	3.663	9.34			1.964
Salts formed on AAM with Silos	6.665	6.758	27.15	2.347		57.02	2.407	

**Figure A1:**
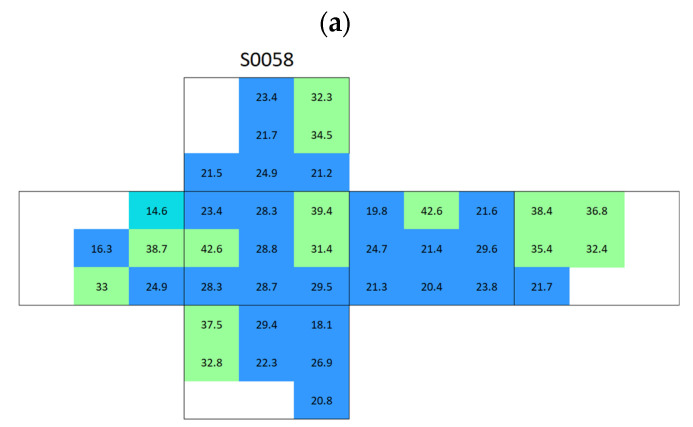

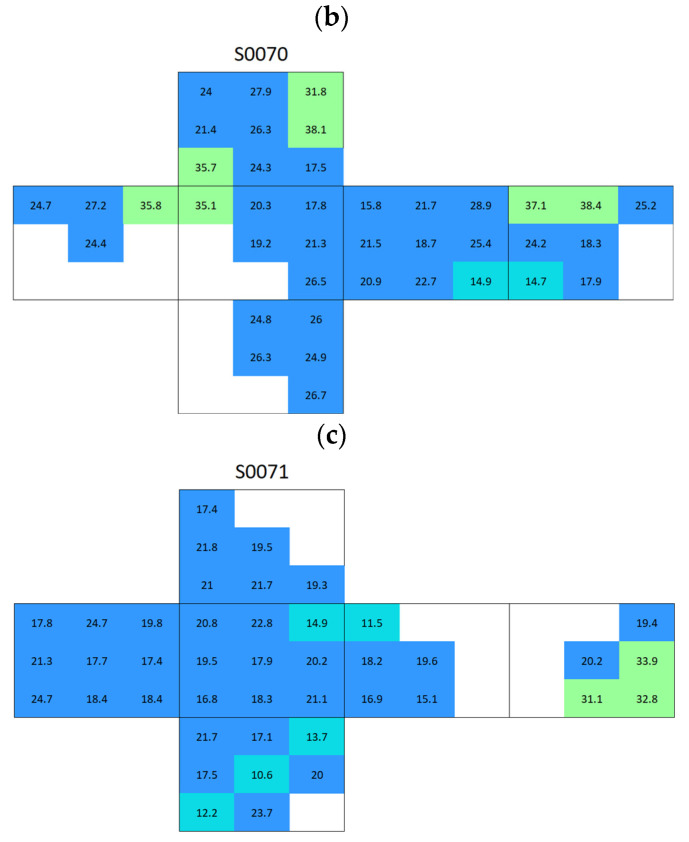
Surface conductivity maps of individual Silos stones: (**a**) S0058, (**b**) S0070, and (**c**) S0071.

**Figure A2:**
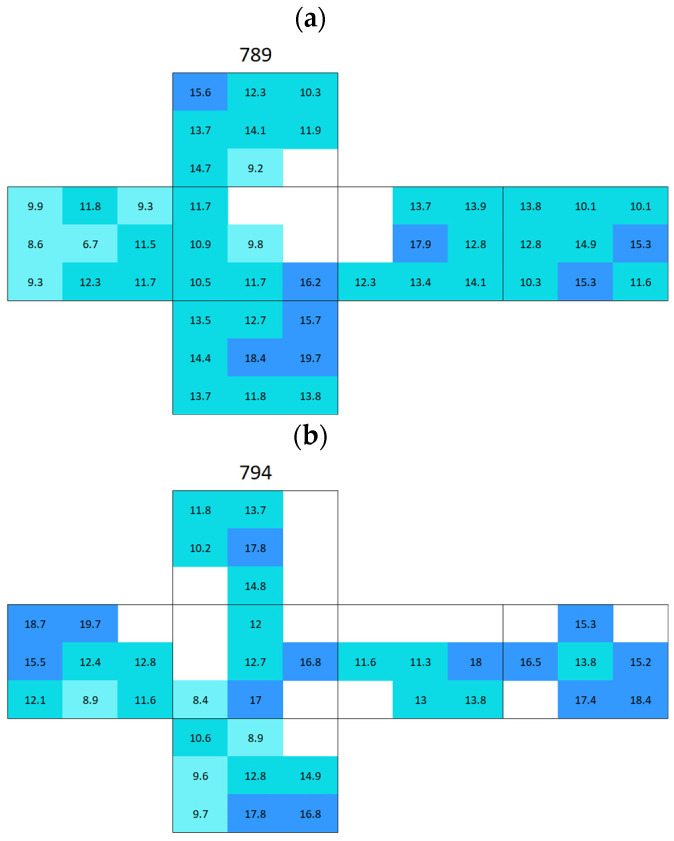

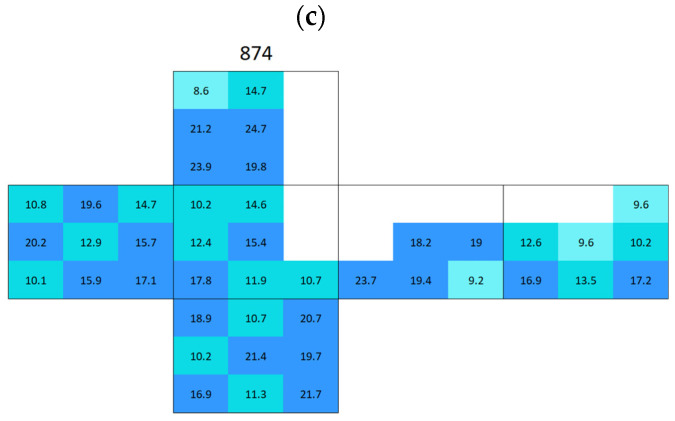
Surface conductivity maps of individual Boñar stones: (**a**) 789, (**b**) 794, and (**c**) 874.

## Data Availability

The original contributions presented in this study are included in the article. Further inquiries can be directed to the corresponding authors.
